# Positive associations between sex hormones, bone metabolism and cognitive impairment in Chinese oldest-old females

**DOI:** 10.1186/s12888-023-04957-9

**Published:** 2023-08-04

**Authors:** Long Feng, Lihua Bian, Chaoxue Ning, Pei Zhang, Yali Zhao, Zhitao Gao, Ping Ping, Shihui Fu

**Affiliations:** 1 Department of Anesthesia, Hainan Hospital of Chinese People’s Liberation Army General Hospital, Sanya, China; 2 Department of Obstetrics and Gynecology, Hainan Hospital of Chinese People’s Liberation Army General Hospital, Sanya, China; 3 Central Laboratory, Hainan Hospital of Chinese People’s Liberation Army General Hospital, Sanya, China; 4 School of Life Science, Beijing Institute of Technology, Beijing, China; 5 School of Laboratory Medicine, Xinxiang Medical University, Xinxiang, China; 6 General Station for Drug and Instrument Supervision and Control, Joint Logistic Support Force of Chinese People’s Liberation Army, Beijing, China; 7 Department of Cardiology, Hainan Hospital of Chinese People’s Liberation Army General Hospital, Sanya, China; 8 Department of Geriatric Cardiology, Chinese People’s Liberation Army General Hospital, Beijing, China

**Keywords:** Bone metabolism, Cognitive impairment, Oldest-old females, Sex hormones

## Abstract

**Purpose:**

With a rapid increase in older adults, progressive impairment in cognitive function has become an increasing concern owing to high social and economic burdens. The current study was designed to investigate the associations of sex hormones and bone metabolism with cognitive impairment (CI) in Chinese oldest-old females.

**Methods:**

There were 396 oldest-old females from the China Hainan Oldest-old Cohort Study (CHOCS). Following standardized procedures, Mini Mental State Examination was effectively completed, and sex hormones and bone metabolism were assessed in these females.

**Results:**

The median age of all females was 101 years (range: from 80 to 116). There were 340 females (86%) with CI. Participants with CI had significantly higher levels of age, progesterone, prolactin and estradiol than those without CI (P < 0.05 for all). Total type I collagen N-terminal elongation peptide [hazard ratio (HR): 1.018, 95%CI: 1.001–1.035] and prolactin (HR: 1.065, 95%CI: 1.005–1.129) levels were positively and significantly associated with CI (P < 0.05 for all).

**Conclusions:**

Prolactin and total type I collagen N-terminal elongation peptide had positive associations with CI in Chinese oldest-old females. Thus, a balance in sex hormones and bone metabolism may have significant effects on cognitive function during the aging process.

## Introduction

Aging is associated with an impairment in cognitive function, including verbal and visual memory, executive function, and spatial ability [1]. The number of older people is estimated to increase from 524 million in 2010 to 1.5 billion in 2050 all over the world [2]. Approximately 4.6 million people are diagnosed with dementia each year, with a 2-fold increase in the disease prevalence every 20 years [3]. Therefore, with a rapid increase in older adults, progressive impairment in cognitive function has become an increasing concern owing to high social and economic burdens [4, 5].

Pre-clinical and clinical studies have demonstrated that older females have a higher prevalence of cognitive impairment (CI), which may be closely connected with sex hormones, especially after menopause [6–8]. Approximately 60% women has CI after menopause, and hormonal change plays important effects on cognitive function [9]. There are several hypotheses on the sex differences in CI, such as difference in brain volume, anatomy and metabolism [10, 11]. Furthermore, there are reduced bone mineral density as a result of aging and physical inactivity. Previous study has indicated that CI have significant relationships with bone mineral density and sex hormone levels in older females [12, 13]. The pathophysiology of osteoporosis may be also related to the occurrence of dementia [14]. The estrogen receptor regulator Raloxifene has been used for the treatment of osteoporosis and may have the ability to improve cognitive function [15]. However, the exact relationships between between sex hormones, bone metabolism and CI still have no clear understanding in Chinese oldest-old females.

Hainan Province is an area with high prevalence of longevity; in fact, it has the highest population density of oldest-old females in China. Oldest-old females may represent a prototype of human longevity and should be considered the best representative [16]. The China Hainan Oldest-old Cohort Study (CHOCS) provides a considerable population-based sample of oldest-old females [17, 18]. For the first time, the current study was designed to investigate the associations of sex hormones and bone metabolism with CI in Chinese oldest-old females. It was hypothesized that CI may be related to sex hormones and bone metabolism in this population.

## Methods

According to the list of oldest-old adults provided by the Department of Civil Affairs of Hainan Province, household survey was conducted on 1863 participants in 18 cities and counties from June 2014 to December 2016. Inclusion criteria: (1) aged ≥ 80 years; (2) residing in Hainan province. Exclusion criteria: (1) male adults; (2) did not complete Mini-Mental State Examination (MMSE) and had missing sex hormones and bone metabolism. Finally, there were 396 oldest-old females aged ≥ 80 years included in this study. The current study was performed in accordance with the principles stated in the Declaration of Helsinki and received the approval from Ethics Committee of Hainan Hospital of Chinese People’s Liberation Army General Hospital (Sanya, Hainan; Number: 301HNLL-2016-01). Informed consent was obtained from all subjects and their legal guardians.

Following standardized procedures, the household survey method was used to collect basic information with interview questionnaires, physical examinations, and blood tests conducted by professional doctors and nurses who could communicate in the local language. No participants received vitamin D, exogenous steroids, or other treatments that could affect their sex hormones and bone metabolism. The primary indicator was MMSE. It is well known that cognitive function was measured using the MMSE [19]. The Georgia Centenarian Study has confirmed that education significantly affect the performance of MMSE [20]. The cutoff points for identifying CI had definite distinction for people who had different levels of education: illiteracy with 17 points, elementary school level with 20 points, and junior high school level with 24 points were identified as CI [21, 22].

Blood samples were collected and transported by trained nurses in chilled bio-transport container (4 °C) to our Central Laboratory within 4 h. Plasma levels of sex hormones and bone metabolism were analyzed with the enzymatic analyses (Roche Products Ltd, Basel, Switzerland) on a fully automatic biochemical autoanalyzer (Cobas c702; Roche Products Ltd, Basel, Switzerland). All analyses were carried out by qualified technicians who were blinded to clinical data.

### Statistical analyses

Data were analyzed using the Statistical Package for Social Sciences Version 17 (Chicago, IL, USA). Data were described using means and standard deviations (continuous variables with normal distribution), medians and interquartile ranges (continuous variables with skewed distribution), and numbers and percentages (categorical variables). Characteristic comparison was performed between participants with and without CI using Student’s t-tests for continuous variables with normal distribution, Mann–Whitney U tests for continuous variables with skewed distribution, and Chi-square tests for categorical variables. Logistic regression analysis was applied to analyze the factors associated with CI adjusted by all variables in Table 1 except MMSE and education level. A two-tailed P < 0.05 was regarded as statistically significant.

**Table 1:** Characteristics of all participants with and without cognitive impairment

Characteristics	With cognitive impairment (n = 340)	Without cognitive impairment (n = 56)	X^2 a^	Z ^b^	P
Age (year)	101 (91, 103)	92 (84, 102)		-3.246	0.001
Body mass index (kg/m^2^)	18 (16,21)	19 (17, 22)		-1.696	0.090
Education level, n (%)			3.607		0.307
Illiteracy	330(97.1)	55(98.2)			
Primary school	1(0.3)	0(0)			
High school	9(2.3)	1(1.8)			
Race, n (%)			1.822		0.610
Han	303(89.1)	53(94.6)			
Others	37(10.9)	3(5.4)			
Diabetes	22(6.5)	5(8.9)	0.457		0.499
Coronary heart disease, n (%)	14(4.1)	3(5.4)	0.180		0.672
Total type I collagen n-terminal elongation peptide (ug/L)	69.0 (52.0, 90.0)	63.0 (50.3, 76.8)		-1.595	0.111
β-crossLaps (ng/ml)	0.34 (0.21, 0.52)	0.31 (0.17, 0.49)		-1.238	0.216
Osteocalcin (ng/ml)	28.9 (19.9, 39.4)	26.8 (18.3, 33.9)		-1.351	0.177
Parathyroid hormone (pg/ml)	43.4 (31.5, 56.2)	38.6 (28.5, 48.8)		-1.890	0.059
25-hydroxyvitamin-D3 (ng/ml)	22.3 (17.2, 27.9)	24.1 (19.8, 28.7)		-1.538	0.124
Phosphonium ion (mmol/L)	1.08 (0.97, 1.18)	1.05 (0.97, 1.19)		-0.425	0.671
Calcium ion (mmol/L)	2.45 (2.16, 2.31)	2.25 (2.18, 2.31)		-0.985	0.325
Progesterone (nmol/L)	0.43 (0.10, 0.89)	0.32 (0.10, 0.57)		-2.372	0.018
Prolactin (ug/L)	12.9 (10.3, 17.6)	10.8 (8.6, 14.4)		-3.341	0.001
Estradiol (pmol/L)	18.4 (18.4, 44.9)	18.4 (18.4, 29.8)		-2.537	0.011
Testosterone (nmol)	0.35 (0.15, 0.63)	0.35 (0.11, 0.73)		-0.175	0.861
Human chorionic gonadotropin (U/L)	2.44 (1.36, 4.00)	2.26 (1.17, 3.36)		-1.576	0.115
Luteinizing hormone (mlU/ml)	36.0(28.1, 45.8)	34.4(28.3, 44.2)		-0.992	0.357
Follicle stimulating hormone (IU/L)	80.8 (62.7, 100.8)	79.3 (63.1, 100.3)		-0.126	0.900
Mini Mental State Examination	8.5 (6.0, 12.0)	21.0 (19.0, 25.0)		-12.016	< 0.001

^a^Chi-square tests; ^b^Mann-Whitney U tests

## Results

The median age of all females was 101 years (range: from 80 to 116). There were 340 females (86%) with CI. Table 1 showed characteristic comparison between all participants with and without CI. Participants with CI had significantly higher levels of age, progesterone, prolactin and estradiol than those without CI (P < 0.05 for all). As shown in Table 2, total type I collagen N-terminal elongation peptide [hazard ratio (HR): 1.018, 95%CI: 1.001–1.035] and prolactin (HR: 1.065, 95%CI: 1.005–1.129) levels were positively and significantly associated with CI (P < 0.05 for all).

**Table 2:** Factors associated with cognitive impairment in Logistic regression analysis of Chinese oldest-old females

Factors	HR	95%CI	P
Age (year)	1.042	1.000—1.086	0.051
Body mass index (kg/m^2^)	0.991	0.909—1.081	0.839
Race, n (%)	2.038	0.604–6.876	0.251
Diabetes, n (%)	0.873	0.286–2.666	0.812
Coronary heart disease, n (%)	0.733	0.181–2.974	0.664
Total type I collagen N-terminal elongation peptide (ug/L)	1.018	1.001—1.035	0.038
β-crossLaps (ng/ml)	0.177	0.027—1.173	0.073
Osteocalcin (ng/ml)	0.981	0.949—1.015	0.269
Parathyroid hormone (pg/ml)	1.017	0.996—1.037	0.107
25-hydroxyvitamin-D3 (ng/ml)	0.988	0.946—1.031	0.578
Phosphonium ion (mmol/L)	2.033	0.377—10.965	0.409
Calcium ion (mmol/L)	2.421	0.090—65.096	0.598
Progesterone (nmol/L)	2.658	0.983—7.187	0.054
Prolactin (ug/L)	1.065	1.005—1.129	0.034
Estradiol (pmol/L)	1.004	0.989—1.020	0.590
Testosterone (nmol)	1.094	0.784—1.527	0.596
Human chorionic gonadotropin (U/L)	1.135	0.936—1.376	0.199
Luteinizing hormone (mlU/ml)	1.002	0.963—1.043	0.909
Follicle stimulating hormone (IU/L)	1.001	0.984—1.018	0.915

HR: hazard ratio; CI: confidence interval

## Discussion

The current study provided epidemiological evidence that prolactin and total type I collagen N-terminal elongation peptide levels were positively and significantly associated with CI in Chinese oldest-old females. All the above results give us a clue that an impairment in cognitive function of oldest-old females may be contributed by the change in sex hormones and bone metabolism.

Sex hormones play important roles in the brain of ovariectomized rodent, suggesting that menopause causes the changed levels of circulating sex hormones, which may have a certain effect on the occurrence of dementia. The current results showed that prolactin levels were positively and significantly associated with CI in Chinese oldest-old females. The Henderson recent review has indicated similar results that prolactin may have deleterious effects on cognitive function in older females after menopause [23]. One large study of women not taking hormone therapy showed significant relationships of prolactin with both verbal memory and global cognition [24]. Besides, a cohort study of Japanese-American women has realized that the users of prolactin showed an impairment in cognitive function [25, 26]. However, prolactin have been reported to play protective effects on pathogenic or brain dysfunction in various cell and animal models, such as different stroke types, traumatic brain injury, and age-related neurodegenerative diseases [27–29]. In a word, there exists definite associations between CI and prolactin, but they are very complex, especially in postmenopausal oldest-old females.

Bone has been established as an endocrine organ regulating energetic metabolism, insulin secretion, and cognitive function [30–33]. Bone metabolism has been identified to affect cognitive function [30]. Besides, low bone mineral density is related to CI in community-dwelling adults [34]. Recent studies have confirmed that circulating biomarkers of bone metabolism may have clinical potential to predict worsening CI [35, 36]. It is well known that bone formation is represented by total type I collagen n-terminal elongation peptide. The current study found that total type I collagen n-terminal elongation peptide levels were positively and significantly associated with CI, suggesting that CI may have positive association with bone resorption. Thus, an improvement in bone health may have beneficial effects on cognitive function during the aging process [37, 38].

Over the past 20–30 years, issues related to aging, multimorbidity, and poly pharmacy have become a prominent problem in healthcare worldwide [39]. In a retrospective study of patients older than 65 years admitted from a home care facility, 66% used five or more medications, 46% used more than seven medications, and 21% used 10 or more medications [40]. The number of drugs per prescription and their age are susceptibility factors for drug drug interaction (DDI) and potential adverse reactions (ADR). The prevalence of DDI and ADR in older adults has been reported to be two to three times greater than that in younger ones [41]. Therefore, poly pharmacy may also contribute to an increase in prolactin levels. An increased risk in older adults may be related to impaired organ reserve capacity, common multiple organ dysfunction, and altered pharmacokinetics and pharmacodynamics [42].

The new contribution of this study was that the study population was very representative. In the long-lived oldest-old females in Hainan Island of China, prolactin and total type I collagen N-terminal elongation peptide were found to have positive associations with CI, which provides scientific basis for cognitive aging prevention and brain health care of older women. However, this study has one limitation: it is a cross-sectional study. No causal relationship can be determined by this study.

## Conclusions

The current study demonstrated that prolactin and total type I collagen N-terminal elongation peptide had positive associations with CI. Thus, a balance in sex hormones and bone metabolism may have significant effects on cognitive function during the aging process.

## Data Availability

All data and material are available under the requirement to the corresponding authors.

## List of abbreviations

CI: cognitive impairment
CHOCS: China Hainan Oldest-old Cohort Study
HR: hazard ratio
MMSE: Mini-Mental State Examination.
